# Functional status associated with postural dizziness, but not postural hypotension, in older adults: a community-based study

**DOI:** 10.1186/s12877-023-04100-z

**Published:** 2023-06-22

**Authors:** Hsiang-Ju Cheng, Zih-Jie Sun, Feng-Hwa Lu, Yi-Ching Yang, Chih-Jen Chang, Jin-Shang Wu

**Affiliations:** 1 Department of Family Medicine, National Cheng Kung University Hospital, College of Medicine, National Cheng Kung University, Tainan, Taiwan; 2 Department of Family Medicine, National Cheng Kung University Hospital Dou-Liou Branch, College of Medicine, National Cheng Kung University, Yunlin, Taiwan; 3 Department of Family Medicine, College of Medicine, National Cheng Kung University, Tainan, Taiwan; 4 Department of Family Medicine, Ditmanson Medical Foundation Chia-Yi Christian Hospital, Chia-Yi, Taiwan

**Keywords:** Postural dizziness, Postural hypotension, ADLs, IADLs, Functional status, Older adults

## Abstract

**Background:**

Functional status, postural dizziness (PD), and postural hypotension (PH) were important issues in older adults. Only one study on the relationship for the three of them in female was without adjusting some important associated factors. This study was intended to investigate the association of PD and PH with functional status in older people of both genders.

**Methods:**

Based on a stratified randomized cluster sampling, 1361 subjects ≥ 65 years in the community were recruited from Tainan City, Taiwan, from 2000 to 2001. PH was defined as a decrease in systolic/diastolic blood pressure of ≥ 20/10 mm Hg after 1 or 2 min of standing. PD was defined by a positive response to dizziness-like symptoms after standing up from a supine position. Functional status included the activities of daily living (ADLs) and instrumental activities of daily living (IADLs).

**Results:**

After adjusting other variables, ADL disability (OR: 1.84, 95% CI: 1.35–2.51) and IADL disability (OR: 1.62, 95% CI: 1.21–2.17) were associated with PD, but not PH. In male and female subgroups, ADL disability (male OR: 1.70, 95% CI: 1.08–2.67; female OR 1.96, 95% CI: 1.26–3.07) was associated with PD. In male, IADL disability was associated with PD (OR: 2.32, 95% CI: 1.36–3.95).

**Conclusions:**

Impaired functional status, shown using ADLs or IADLs, was positively associated with PD, but not PH in older adults ≥ 65 years. Clinically, it may be important to evaluate PD in older adults with ADL or IADL disability.

## Background

Dizziness is commonly associated with the risk of falling and disability in older adults [1, 2]. Postural dizziness (PD) is one manifestation of dizziness. The prevalence of PD increases with age [3]. Although PD was found to be associated with postural hypotension (PH) [4, 5], the factors associated with PD were heterogeneous. Aging, comorbidities (heart failure, diabetes mellitus, stroke, impaired exercise tolerance, and cataract) [3, 6], and medications (anti-anxiety medication, sedatives, hypnotics, seizure medication, and diuretics) [3, 7] are commonly associated with PD. In clinical practice, PH is defined as a reduction in blood pressure in the standing position. Half of the population with PH may self-report dizziness in the past year [8]. PH has been found to be associated with chronic diseases, such as dementia, cardiovascular diseases, cardiac failure, cerebral infarction, kidney disease, diabetes mellitus, Parkinson’s disease, multiple sclerosis, and spinal cord injury [8–11]. Medications, such as alpha blockers, antipsychotics, diuretics, beta blockers, bromocryptine, levodopa, marijuana, narcotics and sedatives, sildenafil, tricyclic antidepressants, and vasodilators, have been associated with PH [12].

In older adults, functional status is considered to be one aspect of health [13]. Even though age is accompanied by comorbidities, to maintain older adults’ functional status is important. People with worse functional status have worse clinical outcomes and higher rates of mortality [14, 15]. Activities of daily living (ADLs) and instrumental activities of daily living (IADLs) are two common tools used to evaluate functional status. ADLs provide a measurement for abilities to meet basic physical needs. The domains of ADLs include bathing, dressing, toileting, transferring, continence, and feeding, and all are used for maintaining living and in care settings [16]. IADLs provide a measurement of independent living activities for those living in the community. The domains of IADLs include using telephone, cooking, cleaning, ability to transporting independently, laundry, shopping, taking own medications, managing finances [17], and all are used for work and social involvement being more complex than that in ADLs. Both ADLs and IADLs are affected by many comorbidities in older people. For example, ADL disability have been associated with cerebrovascular diseases, cognitive impairment, wounds/injuries, and undernutrition [18, 19]. Disabilities related to IADLs have been related to neurological problems, including cerebrovascular diseases, Parkinson’s disease, and cognitive impairment [19].

To the best of our knowledge, studies on the relationships among PD, PH, and functional status were rare [3]. One previous study focused on the relationships between PD and PH among older women [3]. The results showed that PD had stronger association than PH with impaired functional status, as defined by IADLs. However, the above study did not include older males, and ADL disability was not measured. In addition, the above research did not adjust for some important associated factors, such as gender, chronic diseases, medication use, and lifestyle. The aim of this study was to investigate the associations of PD and PH with functional status, including ADLs and IADLs, in older adults of both genders.

## Methods

The cross-sectional data were collected from a decoded database without any personal identification information based on a stratified randomized cluster sampling examination of people ≥ 65 years in Tainan City, which was divided into 7 strata by the administrative districts, for the period from 2000 to 2001. In each stratum, one area was randomly selected by adopting a probability proportional to the size of the areas within that specific stratum. All the subjects aged ≥ 65 years were invited to participate the study. A total of 1361 subjects were included after excluding subjects with heavy drinking (males ≥ 210gm /week and females ≥ 140gm /week, *n* = 42) [20] and missing data (incomplete questionnaire response or test results, *n* = 35). This study was approved by the Institutional Review Board of National Cheng Kung University Hospital, Taiwan (IRB Number: A-ER-106–389).

None the subjects consumed alcohol, coffee, tea, or cigarettes on the day of the examination and underwent history taking and questionnaire, physical examination, PD, PH and blood tests on the same day. Then, the blood analysis result could be collected from medical record. The clinical examination was performed by physicians. The structural questionnaire included age, gender, medical history, medication, lifestyle habits (cigarette smoking, alcohol consumption, and regular exercise), ADLs, and IADLs. A history of cerebrovascular disease, arrhythmia, and ischemic heart disease was reviewed by a doctor. ADL and IADL were evaluated by questionnaire, referring the section of daily activities from Chinese-version Multidimensional Functional Assessment Questionnaire (CMFAQ), Cronbach's α was above 0.70 in each domain of that questionnaire. [21] ADLs included the following domains:1) self-feeding, 2) dressing, 3) grooming, 4) the ability to walk, 5) the ability to get in and out of bed, 6) bathing and showering, and 7) getting to the toilet. Each domain was evaluated using three scales (does not need help, needs partial help, and totally dependent) [21, 22]. IADLs included the following domains: 1) using the telephone, 2) arriving to somewhere by yourself, 3) shopping for groceries and necessities, 4) preparing meals, 5) doing housework, 6) taking prescribed medications, 7) managing money. Each domain was also evaluated using three scales (does not need help, needs partial help, and totally dependent) [21, 22]. One of the seven domains of ADLs or IADLs where the subject needed partial help or was totally dependent was called ADL or IADL disability, respectively [21, 22]. Cigarette smoking was classified as current (defined as at least 1 pack per month for at least half a year) and non-smoker. Alcohol consumption was classified as current (defined as at least once per week for at least half a year) and non-drinker. Regular exercise was defined as exercise with increases in heart rate and breathing for a minimum of 20 min on three or more times weekly [23, 24].

The body mass index (BMI) was calculated as weight in kilograms divided by the square of height in meters. According to the Asian-Pacific cutoff points, obesity was defined as BMI ≥ 25 kg/m2 [25]. Blood pressure and heart rate were measured with a DINAMAP vital sign monitor (Model 1846SX; Critikon, Irvine, USA) with a rest of least 5 min before the morning meal. Hypertension was defined as a positive history of hypertension or resting blood pressure ≥ 140/90 mmHg. After supine blood pressure and heart rate were measured, the subject then stood from the supine position. Blood pressure was recorded from the right forearm, which was relaxed and supported at about heart level by an adjustable table after 1 and 2 min of standing. A positive or negative response to any symptoms, including dizziness, light headedness, or faintness, was recorded after the subjects stood up. PH was defined as a decrease in systolic blood pressure of ≥ 20 mm Hg or diastolic blood pressure of ≥ 10 mm Hg after either 1 or 2 min of standing [7, 26]. PD was defined by a positive response to dizziness-like symptoms after standing up from a supine position. [7] Diabetes mellitus was defined as fasting glucose ≥ 126 mg/dL, hemoglobin A1c (HbA1c) ≥ 6.5% or having a positive history of diabetes. The definition of chronic kidney disease was eGFR < 60 or microalbuminuria ≥ 30 mg/g [27].

### Statistical analyses

We used the 17th version of the SPSS (Chicago, IL, USA) software to perform the statistical analyses. The differences in the clinical characteristics between the groups with or without both PD and PH were assessed using the Student’s t test for continuous variables (presented as mean ± SD) and a chi-squared analysis for the categorical variables (presented as a percentage). P value in univariate analysis and the importance of variables in reference were used to their selection for each model. In the binary logistic regression with a default setting of enter selection, in the total subjects, the association of functional status (independent variable) with PH (dependent variable) and PD (dependent variable) was analyzed, respectively, with adjustment for other variables including age, gender, obesity, hypertension, diabetes mellitus, history of cerebrovascular disease, chronic kidney disease, history of arrhythmia, history of ischemic heart disease, current smoking, mild/moderate alcohol consumption, regular exercise, and used of antidepressants, antipsychotics, and sedative/hypnotics. Then, the association of functional status with PD and PH were examined by gender. Statistical significance was defined as a *p* value < 0.05 throughout the analysis.

## Results

Based on this large transversal study, the prevalence of PD and PH were 30.9% and 20.4%, respectively. The prevalence of people with both PD and PH was 7.3%. Furthermore, the prevalence of ADL disability and IADL disability were 28.3% and 34.3% in subjects with both PD and PH. Table 1 shows that PD was more prevalent in females (*p* < 0.001) and in those with a drop in the diastolic blood pressure after standing 2 min (*p* = 0.007), a history of cerebrovascular disease (*p* = 0.021), ADL and IADL disability (both *p* < 0.001), but with a lower prevalence of smoking (*p* = 0.002) and mild/moderate alcohol consumption (*p* < 0.001) than subjects without PD. As compared to subjects without PH, subjects with PH were older (*p* = 0.004) and had higher supine systolic and diastolic blood pressure (*p* < 0.001 and *p* = 0.001, respectively), fasting plasma glucose (*p* = 0.022), HbA1c (*p* = 0.001), triglyceride (*p* = 0.048), creatinine (*p* = 0.014), and the prevalence of microalbuminuria (*p* = 0.008), diabetes mellitus (*p* = 0.005), chronic kidney disease (*p* < 0.001), a history of cerebrovascular disease (*p* = 0.019), a history of ischemic heart disease (*p* = 0.002), ADL disability (*p* = 0.012), and current smoking (*p* = 0.001). Besides, subjects with PH exhibited a drop in the systolic and diastolic blood pressure after standing 1 min and 2 min.

**Table 1:** Comparison of clinical variables between subjects with and without postural dizziness (PD) and postural hypotension (PH)

	PD			PH		
	No (*n* = 937)	Yes (*n* = 424)	*p* value	No (*n* = 1083)	Yes (*n* = 278)	*p* value
Age, years^a^	71.8 ± 4.9	72.2 ± 5.3	0.259	71.7 ± 5.0	72.7 ± 5.2	0.004
Female^b^	40.0	58.0	< 0.001	48.7	33.8	< 0.001
Body mass index, kg/m^2a^	24.6 ± 3.5	24.5 ± 3.6	0.822	24.6 ± 3.5	24.4 ± 3.7	0.563
Supine SBP, mmHg^a^	136.3 ± 23.0	135.5 ± 23.9	0.562	134.6 ± 23.1	141.8 ± 23.4	< 0.001
Supine DBP, mmHg^a^	73.7 ± 11.9	72.8 ± 12.1	0.202	72.9 ± 11.9	75.5 ± 11.9	0.001
SBP drop after standing 1 min, mmHg^a^	0.8 ± 15.8	1.3 ± 19.2	0.672	-4.2 ± 13.2	21.3 ± 14.9	< 0.001
DBP drop after standing 1 min, mmHg^a^	-2.3 ± 8.2	-2.7 ± 9.2	0.451	-4.7 ± 7.0	6.3 ± 8.2	< 0.001
SBP drop after standing 2 min, mmHg^a^	-2.7 ± 15.3	-3.3 ± 19.3	0.651	-7.5 ± 13.8	14.7 ± 15.1	< 0.001
DBP drop after standing 2 min, mmHg^a^	-2.6 ± 8.0	-3.9 ± 9.3	0.007	-5.1 ± 7.2	5.1 ± 8.0	< 0.001
FPG, mg/dl^a^	106.8 ± 38.2	109.5 ± 42.4	0.245	106.2 ± 37.7	113.0 ± 46.0	0.022
2-h PG, mg/dl^a^	151.0 ± 65.1	152.9 ± 68.9	0.659	151.5 ± 65.2	152.5 ± 70.8	0.937
HbA1c, %^a^	5.8 ± 1.5	5.9 ± 1.7	0.676	5.8 ± 1.5	6.1 ± 1.8	0.001
Cholesterol, mg/dl^a^	207.3 ± 40.8	209.5 ± 41.6	0.353	207.4 ± 40.3	210.2 ± 44.0	0.313
Triglyceride, mg/dl^a^	131.0 ± 73.2	139.8 ± 87.5	0.060	131.1 ± 73.3	142.9 ± 91.2	0.048
HDL-C, mg/dl^a^	49.7 ± 14.6	49.7 ± 15.2	0.994	50.0 ± 14.7	48.6 ± 15.1	0.173
Creatinine, mg/dl^a^	1.1 ± 0.6	1.0 ± 0.6	0.153	1.0 ± 0.7	1.1 ± 0.5	0.014
Microalbuminuria^b^	37.9	41.6	0.200	37.3	46.0	0.008
Hypertension^b^	67.7	70.0	0.381	67.9	70.5	0.399
PH^b^	19.1	23.3	0.072	-	-	-
PD^b^	-	-	-	30.0	35.6	0.072
Diabetes mellitus^b^	24.7	24.1	0.813	22.8	30.9	0.005
Chronic kidney disease^b^	61.2	61.3	0.953	58.8	70.5	< 0.001
History of cerebrovascular disease^b^	3.9	6.8	0.021	4.2	7.6	0.019
History of arrhythmia^b^	6.5	4.2	0.098	5.4	7.2	0.267
History of ischemic heart disease^b^	6.6	8.5	0.216	6.1	11.5	0.002
ADL disability^b^	13.6	24.3	< 0.001	15.6	21.9	0.012
IADL disability^b^	18.7	33.7	< 0.001	23.1	24.8	0.520
Mild/moderate alcohol consumption^b^	10.8	4.0	< 0.001	8.3	10.1	0.352
Current smoking^b^	27.6	19.8	0.002	23.2	33.1	0.001
Regular exercise^b^	6.4	6.1	0.849	6.1	7.2	0.501
Antidepressants use^b^	1.5	2.6	0.162	1.8	2.2	0.655
Antipsychotics use^b^	4.5	6.6	0.101	4.9	6.1	0.411
Sedative/hypnotics use^b^	7.3	8.0	0.621	7.7	6.8	0.639

Data presented as mean ± stand deviation or percentile

*SBP* Systolic blood pressure, *DBP* Diastolic blood pressure, *FPG* Fasting plasma glucose, *2-h PG* 2-h post-load glucose, *HbA1c* Hemoglobin A1c, *HDL-C* High-density lipoprotein cholesterol, *ADL* Activities of daily living, *IADL* Instrumental activities of daily living

^a^Student’s t test

^b^Chi-squared test

Table 2 reveals the results of the logistic regression analysis for the relationships between the clinical variables and PD. It showed that female gender (*p* < 0.001, OR = 1.76, 95% CI: 1.31–2.37), PH (*p* = 0.045, OR = 1.36, 95% CI: 1.01–1.83), ADL disability (*p* < 0.001, OR = 1.84, 95% CI: 1.35–2.51), IADL disability (*p* = 0.001, OR = 1.62, 95% CI: 1.21–2.17), and mild/moderate alcohol consumption (*p* = 0.003, OR = 0.42, 95% CI: 0.24–0.75) were significantly associated with PD. Table 3 shows the logistic regression analysis for the association between the clinical variables and PH. Female gender (*p* = 0.001, OR = 0.56, 95% CI: 0.40–0.79), PD (*p* = 0.046, OR = 1.35, 95% CI: 1.01–1.82), diabetes mellitus (*p* = 0.048, OR = 1.37, 95% CI: 1.00–1.86), chronic kidney disease (*p* = 0.018, OR = 1.45, 95% CI: 1.06–1.96), and a history of ischemic heart disease (*p* = 0.020, OR = 1.73, 95% CI: 1.09–2.75) were independently related to PH. However, ADL and IADL disability were not related to PH.

**Table 2:** Results for the binary logistic regression analysis of the relationships between the clinical variables and postural dizziness

	Odds ratio	(95% CI^c^)	*p* value
Age, years	1.00	(0.98–1.03)	0.903
Body mass index ≥ 25 kg/m^2^, yes = 1, no = 0	1.08	(0.84–1.39)	0.536
Female = 1 vs. male = 0	1.76	(1.31–2.37)	< 0.001
Postural hypotension, yes = 1, no = 0	1.36	(1.01–1.83)	0.045
Hypertension, yes = 1, no = 0	1.03	(0.79–1.36)	0.812
Diabetes mellitus, yes = 1, no = 0	0.85	(0.63–1.13)	0.261
Chronic kidney disease, yes = 1, no = 0	0.98	(0.76–1.27)	0.869
History of cerebrovascular disease, yes = 1, no = 0	1.55	(0.90–2.67)	0.111
History of arrhythmia, yes = 1, no = 0	0.63	(0.36–1.10)	0.103
History of ischemic heart disease, yes = 1, no = 0	1.24	(0.79–1.95)	0.345
ADL disability, yes = 1, no = 0	1.84	(1.35–2.51)	< 0.001
IADL disability, yes = 1, no = 0	1.62	(1.21–2.17)	0.001
Current smoking, yes = 1, no = 0	1.02	(0.73–1.45)	0.893
Mild/moderate alcohol consumption, yes = 1, no = 0	0.42	(0.24–0.75)	0.003
Regular exercise, yes = 1, no = 0	1.21	(0.74–2.00)	0.444
Antidepressant use, yes = 1, no = 0	1.41	(0.54–3.72)	0.486
Antipsychotic, yes = 1, no = 0	1.55	(0.87–2.77)	0.140
Sedative/hypnotics, yes = 1, no = 0	0.87	(0.53–1.45)	0.594

*ADL* Activities of daily living, *IADL* Instrumental activities of daily living, *CI* Confidence interval

**Table 3:** Results for the binary logistic regression analysis of the relationships between the clinical variables and postural hypotension

	Odds ratio	(95% CI^c^)	*p* value
Age, years	1.02	(0.99–1.05)	0.138
Body mass index ≥ 25 kg/m^2^, yes = 1, no = 0	0.82	(0.62–1.08)	0.160
Female = 1 vs. male = 0	0.56	(0.40–0.79)	0.001
Postural dizziness, yes = 1, no = 0	1.35	(1.01–1.82)	0.046
Hypertension, yes = 1, no = 0	1.06	(0.77–1.45)	0.724
Diabetes mellitus, yes = 1, no = 0	1.37	(1.00–1.86)	0.048
Chronic kidney disease, yes = 1, no = 0	1.45	(1.06–1.96)	0.018
History of cerebrovascular disease, yes = 1, no = 0	1.27	(0.71–2.25)	0.424
History of arrhythmia, yes = 1, no = 0	1.20	(0.70–2.07)	0.513
History of ischemic heart disease, yes = 1, no = 0	1.73	(1.09–2.75)	0.020
ADL disability, yes = 1, no = 0	1.26	(0.88–1.79)	0.203
IADL disability, yes = 1, no = 0	1.17	(0.82–1.67)	0.387
Current smoking, yes = 1, no = 0	1.22	(0.87–1.72)	0.257
Mild/moderate alcohol consumption, yes = 1, no = 0	0.94	(0.58–1.53)	0.794
Regular exercise, yes = 1, no = 0	1.15	(0.68–1.97)	0.603
Antidepressant use, yes = 1, no = 0	1.06	(0.35–3.22)	0.922
Antipsychotic, yes = 1, no = 0	1.31	(0.67–2.56)	0.435
Sedative/hypnotics, yes = 1, no = 0	0.74	(0.40–1.36)	0.333

*ADL* Activities of daily living, *IADL* Instrumental activities of daily living, *CI* Confidence interval

The analytical results for the subgroup by gender indicating the relationships between functional status and PD are shown in Table 4. In both genders, PH was not related to PD. In females, ADL disability was significantly associated with PD (*p* = 0.003, OR = 1.96, 95% CI:1.26 – 3.07), and IADL disability was not related to PD (*p* = 0.100, OR = 1.34, 95% CI: 0.95 – 1.91). In males, both ADL disability (*p* = 0.022, OR = 1.70, 95% CI: 1.08 – 2.67) and IADL disability (*p* = 0.002, OR = 2.32, 95% CI: 1.36 – 3.95) were significantly related to PD.

**Table 4:** Association of functional status with postural dizziness (PD) and postural hypotension (PH) in the elderly by gender based on the binary logistic regression^a^

	Outcome = PD		Outcome = PH	
	Model 1	Model 2	Model 3	Model 4
	Female	Male	Female	Male
	OR (95% CI)	OR (95% CI)	OR (95% CI)	OR (95% CI)
PH	1.42(0.90–2.25)	1.30(0.87–1.93)	-	-
PD	-	-	1.43(0.90–2.28)	1.30(0.88–1.94)
ADL disability	1.96(1.26–3.07)^**^	1.70(1.08–2.67)^*^	1.57(0.89–2.76)	1.11(0.70–1.76)
IADL disability	1.34(0.95–1.91)	2.32(1.36–3.95) ^**^	1.23(0.76–1.99)	1.11(0.64–1.94)

^a^Adjusted age, obesity, hypertension, diabetes mellitus, chronic kidney disease, history of cerebrovascular disease, history of arrhythmia, history of ischemic heart disease, current smoking, mild/moderate alcohol consumption, regular exercise, antidepressant use, antipsychotic use, and sedative/hypnotics use

*ADL* Activities of daily living, *IADL* Instrumental activities of daily living, *OR* Odds ratio, *CI* Confidence interval

*p* value: ^*^ < 0.05, ^**^ < 0.01

## Discussion

To the best of our knowledge, there have been no studies investigating the relationships among PD, PH, and ADLs/IADLs concomitantly in an older population. In this large transversal study, the association of ADL and IADL disability with PD and PH in both older genders was controlling many confounding factors, including age, important chronic diseases, lifestyle habits, and medication use. The findings of this study indicated that impaired functional status, manifested by ADL or IADL disability, was positively associated with PD, but not PH in total subjects. The older adults with either ADL or IADL disability may be more likely to have PD. When considering gender differences, ADL and IADL disability were also positively associated with PD in older men. In older women, ADL disability was positively associated with PD, although ADL disability was not in multivariate analysis. In Ensrud KE et al.’s study, PD was associated with IADL disability in older women after adjusting age [3]. In this study, if we adjusted age only accordingly, PD was also associated with IADL disability in older women. However, the relationship between ADL/IADL disability and PH was insignificant in total, male, and female subjects. Based on the findings, it may be important to evaluate self-reported dizziness after standing up from a supine position in older adults with ADL/IADL disability in clinical practice. The care giver will take the caution in caring the older adults with either ADL or IADL disability, especially concomitantly with PD.

An explanation for the positive link between functional disabilities and PD has not been made clear. ADL/IADL disability and PD may have reciprocal associations because limited activity resulting from ADL/IADL disability may increase orthostatic intolerance due to extensive venous pooling related to less action of the leg skeletal muscle pump [10]. Dizziness in older individuals leaded to their becoming less physically active, in turn contributing to worse lower extremity functions [28]. Some domains of ADLs involvement in postural change included the ability to walk, the ability to get in and out of bed, bathing and showering, and toilet hygiene. Some domains of IADLs, including cleaning and maintaining the house, moving within the community, preparing meals, and shopping for groceries and necessities, involved a postural change to complete the task. In addition, older people with dizziness have been shown to have worse gait speed, stair climbing, and one leg stance [28]. Balance changes with aging might be related to loss of sensory elements and a decline in the ability to integrate information and issue motor commands [29]. The above explanation may explain a link between ADL/IADL disability and PD.

Some factors associated with PD include PH, cerebral hypoperfusion, vestibular dysfunction, visual impairment, and disorders of the proprioceptive system [5, 30–32]. In this study, ADL disability was still related to PD in both genders after adjusting for PH and cerebrovascular disease. There was a positive relationship between IADL disability and PD in the male subjects. This may indicate that mechanisms other than PH and cerebrovascular disease linking ADL/IADL disability and PD may be involved. These links may be related to the integration of vestibular dysfunction, visual impairment, disorders in the proprioceptive system, followed by outputs to musculoskeletal system, maintained postural stability [31, 33]. Vestibular dysfunction has been shown to be associated with greater ADL disability and to be female predominant [34]. Limited functional status due to declining visual acuity and limited ADLs have also been found to be related to dizziness [35]. However, the exact causes have not been fully elucidated, and more studies are needed to clarify the underlying mechanism for further associations between functional disabilities and PD.

Older women were more likely to have PD, which concurred with the findings of a previous study [7]. Older women have reported a higher prevalence of dizziness/imbalance than older men [28]. This finding may be related to gender differences in orthostatic intolerance. As compared to males, females have greater parasympathetic modulations, greater decreases in baroreflex sensitivity, and greater increases in sympathetic activity [36]. This may partially explain why older women with PD may be more sensitive to postural changes and in turn may be more likely to have PD. In this study, to our surprise, mild/moderate alcohol consumption was inversely related to PD. This result should be interpreted with caution, however, although one previous study showed that female subjects above 40 years old who did not consume alcohol were more affected by dizziness than men [35]. One explanation may be that some people engage in less activity, including standing from the supine or sitting positions. Although autonomic dysfunction occurred in approximately 16–73% of chronic and excessive alcohol abusers, PD was rare [37]. In this study, heavy drinkers were excluded due to the limited case number (*n* = 42) and the interfering effect related to the classification of alcohol consumption. Determining the mechanism for the relationship between alcohol consumption and PD thus requires additional investigation.

Impairments in the heart rate response, stroke volume, and vascular resistance during postural changes may lead to PH [12]. In this study, PD was found to be associated with PH; however, the relationship between them was heterogeneous in previous studies [3, 4, 6]. Thus, determining whether the link between PD and PH involves the same risk factors, such as cerebral hypoperfusion, requires further study [3, 32]. Older women were inversely associated with PH, but age was not associated with PH in the present study. The mechanisms related to gender difference have not been fully elucidated. The result of this study was consistent with one study that showed women ≥ 75 years were less prevalent in PH than men ≥ 75 years [38], although there was also a less effective compensation for postural change-induced blood pressure drops in the female group [39]. Because the study confined to the elderly subjects ≥ 65 years was rare for the gender difference on PH [38], more studies are needed in the future. Although studies showed that advancing age was related to PH [3, 8, 40], a recent meta-analysis study, including 24,967 participants aged ≥ 60 years from 20 studies, found that age was not associated with an increased prevalence of PH (*p* = 0.264) [41]. The subjects in this study were confined to persons aged ≥ 65 years. Age was not significantly related to PH. Whether the associated risk was not significantly different in persons aged ≥ 65 years requires further investigation, although aging-related change in activity in the renin-angiotensin system with lower plasma renin, angiotensin, and aldosterone levels leaded to less conservation of salt and water [12, 42]. Diabetes mellitus, chronic kidney disease, and ischemic heart disease were an independently associated factor related to PH in this study, consistent with the findings of previous studies [4, 8, 12]. In patients with diabetes, the related mechanism was mainly related to cardiovascular autonomic neuropathy [43]. In the case of chronic kidney disease, the ability of the kidney to conserve salt and water decline with age, especially during periods of fluid restriction or volume loss [12]. It was known that arterial stiffness leaded to impaired renal perfusion, and postural homeostasis played a role in renal damage, in turn enhancing the risk of PH [44, 45]. In addition, reduced hemoglobin in chronic kidney disease was a potential contributor to PH [46]. For ischemic heart disease, the impaired diastolic filling from aged heart leaded the reduction of stroke volume when preload decreased due to standing or volume contraction [12]. This may partially explain the association between ischemic heart disease and PH in older individuals.

This study had some limitations. First, a causal relationship between functional status disability and PD cannot be established because this study was cross-sectional. Second, the diagnosis of dizziness was from a subjective self-report after a postural change. Thus, chronic repeated episodes, the frequency, duration and severity of dizziness could not be measured. However, we measured PD and PH simultaneously when the subjects actively stood up, which potentially presented a more accurate relationship between PH and PD. Third, although we adjusted age, obesity, many chronic diseases, smoking, mild/moderate alcohol consumption, regular exercise, and medication use to reduce the degree of bias, the influences of vestibular disease and rare neurodegenerative disorder, such as multiple system atrophy, which were not collected in this study, cannot be completely ruled out. We also noted ADL/IADL were associated with increased ORs of PD/PH, which was independent of the selected potential confounders. These potential confounders are potential associated factors for PD/PH and are also considered to have associations with ADL/IADL. Fourth, the subjects were confined to a Taiwanese population, so more studies should be conducted in other ethnic groups.

## Conclusions

In this large transversal study, the results indicate that impaired ADLs/IADLs was positively associated with PD, but not PH. In clinical practice, health care professionals should pay attention to assessing whether there is a problem of self-reported dizziness in patients with disabilities. It may be important to evaluate self-reported dizziness among older adults with ADL/IADL disability after they stand up from a supine position. The care giver will take the caution in caring the older adults with either ADL or IADL disability, especially concomitantly with PD. In addition, older adults with PD must be checked for their functional status. In addition, this study provided a further research direction for underlying mechanism between functional status impairment and PD.

## Data Availability

The data may be available from the corresponding author on reasonable request.

## Abbreviations

PD: Postural dizziness
PH: Postural hypotension
ADL: Activities of daily living
IADLs: Instrumental activities of daily living
OR: Odd ratios
CI: Confidence interval
BMI: Body mass index
SD: Standard deviation
HbA1c: Hemoglobin A1c
SBP: Systolic blood pressure
DBP: Diastolic blood pressure
FPG: Fasting plasma glucose
2-h PG: 2-H post-load glucose
HDL-C: High-density lipoprotein cholesterol
